# The development of a comprehensive toolbox based on multi-level, high-throughput screening of MOFs for CO/N_2_ separations†

**DOI:** 10.1039/d1sc01588e

**Published:** 2021-08-11

**Authors:** Nakul Rampal, Abdulmalik Ajenifuja, Andi Tao, Christopher Balzer, Matthew S. Cummings, Arwyn Evans, Rocio Bueno-Perez, David J. Law, Leslie W. Bolton, Camille Petit, Flor Siperstein, Martin P. Attfield, Megan Jobson, Peyman Z. Moghadam, David Fairen-Jimenez

**Affiliations:** The Adsorption & Advanced Materials Laboratory (A^2^ML), Department of Chemical Engineering & Biotechnology, University of Cambridge Philippa Fawcett Drive Cambridge CB3 0AS UK df334@cam.ac.uk; Department of Chemical Engineering and Analytical Science, The University of Manchester Oxford Road Manchester M13 9PL UK; Centre for Nanoporous Materials, Department of Chemistry, The University of Manchester Oxford Road Manchester M13 9PL UK; Barrer Centre, Department of Chemical Engineering, Imperial College London, South Kensington Campus London SW7 2AZ UK; bp Chemicals Limited Saltend Hull HU12 8DS UK; bp International Limited Chertsey Road, Sunbury-upon-Thames TW16 7BP UK; Department of Chemical and Biological Engineering, University of Sheffield Sheffield S1 3JD UK

## Abstract

The separation of CO/N_2_ mixtures is a challenging problem in the petrochemical sector due to the very similar physical properties of these two molecules, such as size, molecular weight and boiling point. To solve this and other challenging gas separations, one requires a holistic approach. The complexity of a screening exercise for adsorption-based separations arises from the multitude of existing porous materials, including metal–organic frameworks. Besides, the multivariate nature of the performance criteria that needs to be considered when designing an optimal adsorbent and a separation process – *i.e.* an optimal material requires fulfillment of several criteria simultaneously – makes the screening challenging. To address this, we have developed a multi-scale approach combining high-throughput molecular simulation screening, data mining and advanced visualization, as well as process system modelling, backed up by experimental validation. We have applied our recent advances in the engineering of porous materials' morphology to develop advanced monolithic structures. These conformed, shaped monoliths can be used readily in industrial applications, bringing a valuable strategy for the development of advanced materials. This toolbox is flexible enough to be applied to multiple adsorption-based gas separation applications.

## Abstract 2

The separation of challenging mixtures through adsorption is a multidimensional problem that requires a holistic approach. Our toolbox combines experiments, molecular and process simulations with data visualization to find optimal, porous materials.

## Graphical abstract

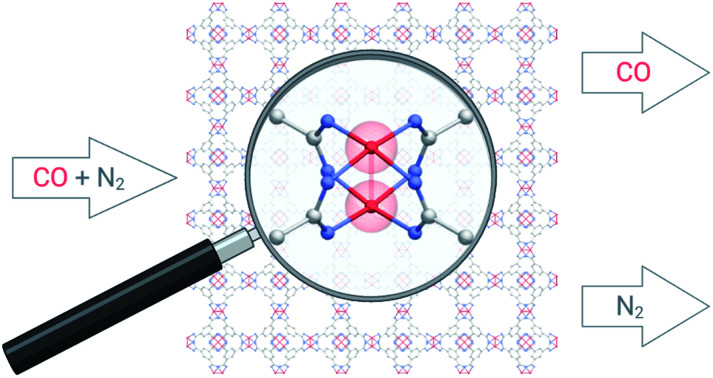

## 1. Introduction

The second law of thermodynamics states that all processes take place so as to increase the entropy of the process. In other words, naturally occurring processes tend to become mixing processes, making separations one of the most challenging tasks in the process industries. As reported by Sholl and Lively,^1^ processes involved in the separation of valuable chemical feedstocks account for 10–15% of the world's energy consumption and, therefore, purifying chemicals through low-emission routes and without energy-intensive processes such as distillation is an urgent demand. In this context, the development of novel adsorbents for gas purification is key for the chemical and petrochemical industries. The premise of this work is that breakthrough improvements of adsorption-based separation processes will require simultaneous efficient screening and development of conformed and engineered materials.

In the past few decades, significant developments of new adsorbents and new processes have been explored in the chemical and petrochemical industries.^2^ Among these, metal–organic frameworks (MOFs), discovered and developed in the last 20 years, have generated great interest from the research and industry community.^3^ MOFs are synthesized *via* the self-assembly of metal ions or clusters and organic linkers.^4^ MOFs show record-high porosity with BET areas as high as ∼8000 m^2^ g^−1^,^5^ as well as high tunability of the textural properties and surface chemistry. Furthermore, some MOFs have coordinated unsaturated metal sites, also called open metal sites (OMS), which have the potential to be used as specific binding sites, making them interesting candidates for CO/N_2_ separations.^6^ Given the large number of existing MOFs, with more than 100 000 MOFs catalogued in the Cambridge Structural Database (CSD) MOF subset to date,^7,8^ a simple trial and error approach is not practical nor fast enough to identify promising structures for chemical separations. To speed up the identification of optimal structures, supercomputers are often used to efficiently screen large number of materials before experimental testing – a technique known as high-throughput screening (HTS). Our recent advances in HTS allow us to apply grand canonical Monte Carlo (GCMC) simulations to rapidly screen *in silico* the adsorption properties of thousands of MOFs, as well as to integrate advances in data mining using bespoke, interactive visualization tools.^9–12^ This screening approach has allowed us to quickly identify top-performing MOFs and to develop structure–property relationships, hence guiding the synthesis of MOFs as industrially advanced adsorbents.^11–14^

Looking at key applications, carbon monoxide is an important and valuable raw material in the chemical industry,^15,16^ being, together with methanol, a key feedstock of the CATIVA™ acetic acid production process.^17^ Whilst methanol is globally traded, CO must be manufactured locally. Current industrial technologies that produce CO – where a purity >99 mol% is desired – involve its purification from synthesis gas (syngas), mainly made up of CO, H_2_, N_2_ and CH_4_, which is produced by either the steam reforming or partial oxidation of natural gas or other fossil fuels (*e.g.* naphtha, fuel oil and coal). To obtain CO, the syngas mixture must be separated. In industry, cryogenic vapour–liquid separation techniques are widely used. Here, conventional cryogenic separation technology is restricted to feeds with low N_2_ content, as associated operating and capital costs become prohibitive as the feed N_2_ content increases. Distillation, which operates based on exploiting differences in the volatility of the components to be separated is not very effective for separating CO/N_2_ mixtures, as they have similar physical properties, with boiling points of 82 K (CO) and 77 K (N_2_), respectively, and hence their separation requires high energy input. Despite the importance of CO/N_2_ separations, and to the best of our knowledge, there are not any robust technologies, currently proven and operating, that can perform the purification process at relatively high N_2_ content at a commercial scale.^18–20^

We study here the potential of MOFs as adsorbents in different cyclic adsorption processes for CO/N_2_ separation. First, we use GCMC simulations to screen existing MOFs and to predict their CO and N_2_ adsorption performance. We then use experimental synthesis and testing to validate the simulation approach. While in single-component adsorption processes, *e.g.* H_2_ storage in a tank, it is clear that an optimal material is often the one with higher adsorption or working capacity (*i.e.* the difference in total uptake between adsorption, *N*^ads^_CO_, and desorption, *N*^des^_CO_, conditions), the situation becomes more complex in multi-component processes, since both working capacity and selectivity are important. In the past, different approaches have been used in materials selection. For example, Llewellyn *et al.*^21^ proposed a series of adsorbent performance indicators (API) based on selectivity, working capacity and adsorption enthalpy for porous materials in separation processes; similarly, Snurr *et al.* used an adsorbent performance score (APS) based on working capacity and selectivity.^22^ To identify the optimal materials here, we investigate a number of performance metrics including CO uptake, CO/N_2_ selectivity, working capacity and adsorbent regenerability. However, in addition to molecular-level simulations, we went one step further by applying a three-step process modelling^23–25^ to explore different practical separation conditions and to optimize process conditions, namely pressure and temperature, for CO/N_2_ separation. This holistic approach has allowed us to build a comprehensive strategy/toolbox to find optimal materials and process conditions for high-performance adsorptive separation of gases coupled with dynamic data visualization, and in particular for MOFs in CO/N_2_ separations. We complement the computational screening work by using recent advances in engineering and densification of MOFs to produce conformed structures readily applicable in industry, reaching volumetric capacities 3–4 times higher than standard powder MOFs.^26^

## 2. Force field modification

**Fig. 1:**
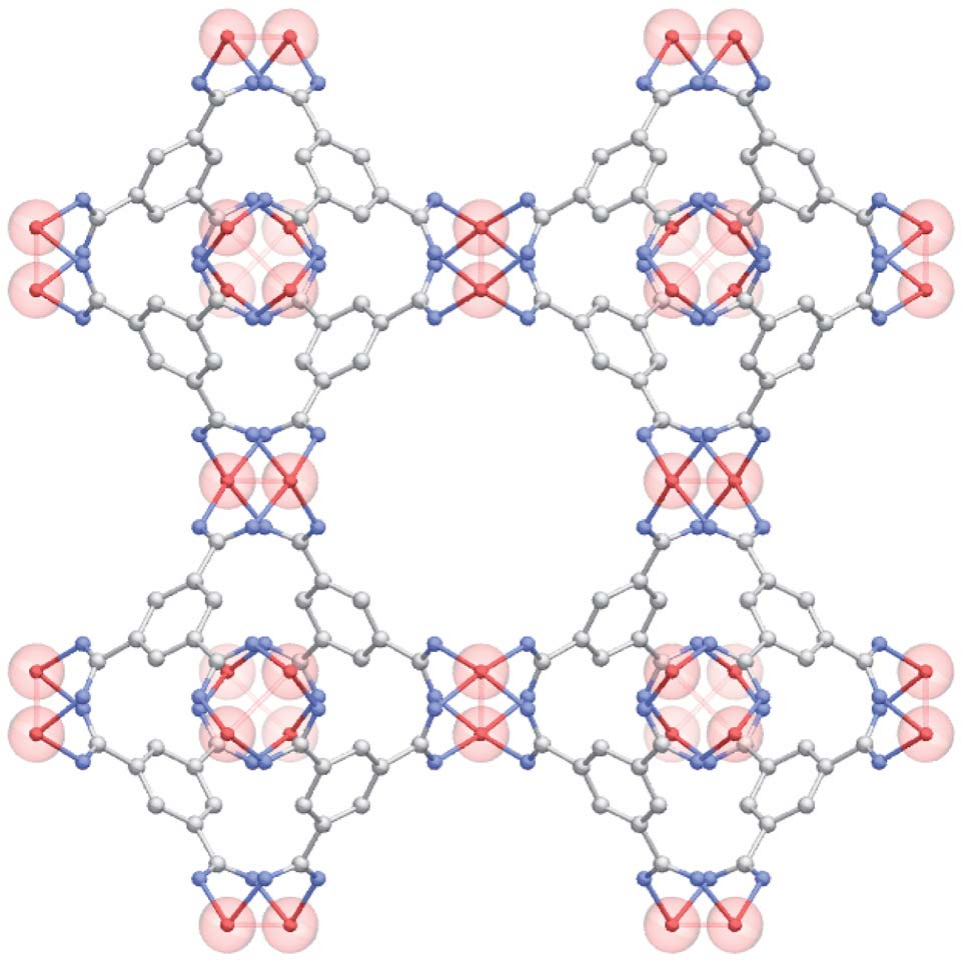
Structure of HKUST-1 with the Cu–Cu paddle-wheels highlighted in red. C atoms in grey; O atoms in blue; Cu atoms in red; H atoms have been omitted for clarity.

Among existing MOFs, previous literature points to Cu–Cu paddle-wheel building units, such as those included in HKUST-1 (Fig. 1), as a structural feature that favors CO adsorptive separation.^6,7,27–30^ This is due to the existence of OMS that can interact preferentially with the CO molecule instead of N_2_; obviously, this is a transition from more generic physisorption towards more selective chemisorption. Looking at MOFs with Cu–Cu paddle-wheels present in the CSD,^7^ we found 183 structures. These structures have pore volumes ranging from 0.13 to 7.55 cm^3^ g^−1^ and volumetric surface areas that span from *ca.* 400 to 2700 m^2^ cm^−3^. We then carried out GCMC simulations on this subset of materials to explore their performance on the selective adsorption of CO over N_2_. Generic force fields such as DREIDING force field (DFF)^31^ and Universal Force Field (UFF)^32^ are widely used in HTS studies because they are transferable and have demonstrated good accuracy for the prediction of adsorption isotherms^33,34^ However, the strong interactions of some gases with the open metal sites due to chemisorption processes, such as that of CO, are not correctly described by these general-purpose force fields and, therefore, need to be modified to provide a more realistic description.^35–37^

**Fig. 2:**
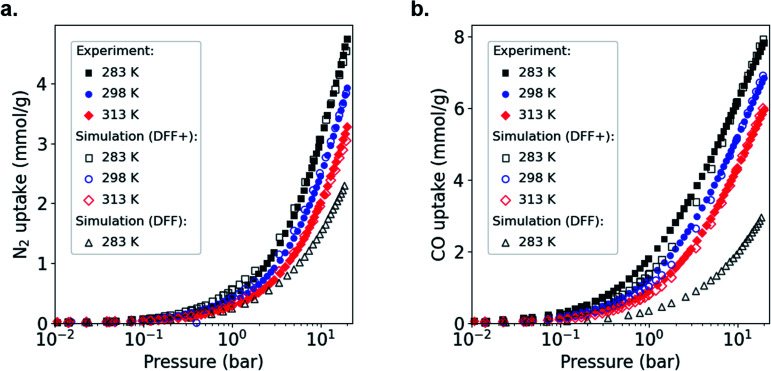
Experimental and simulated adsorption isotherms for (a) N_2_ and (b) CO in _powd_HKUST-1 at different temperatures, 313 K, red diamonds; 298 K, blue circles; and 283 K, black squares. Filled symbols, experimental data; open symbols, DFF+ simulated data. Black triangles represent DFF simulated data at 283 K.

To adapt the generic DFF to MOFs containing open metal sites, we focus on HKUST-1 as the archetypical Cu–Cu paddle-wheel MOF.^6,34,38^ Following our previous approach, we used experimental adsorption data for CO and N_2_ as a reference and then we artificially strengthen the metal-adsorbate interactions in our simulation.^36^ Before modifying force fields using experimental adsorption isotherms, one needs to account first for the fact that experimental samples can include non-porous defects or activation issues, which complicates the comparison with GCMC simulations on perfect crystals.^36,39,40^ Thus, we compared the total adsorbed amount of our experimental and simulated isotherms for N_2_ adsorption in HKUST-1 at 77 K (Fig. S6†), which allows calculation of the experimentally accessible pore volume. Both isotherms are Type I, typical of microporous materials, with a clear plateau due to the saturation of the micropore volume and the nonexistence of mesoporosity. This comparison also shows that the simulated data overestimates the total adsorbed amount from experiments, pointing to non-porous defects in the HKUST-1 sample and, consequently, a reduced total pore volume. Thus, we applied a scaling factor of *ϕ* = 0.85 to match the experimental N_2_ capacity. We would like to point out here that: (i) the scaling factor derived is specific to our samples, and may be different for samples synthesized by another lab, and (ii) the force field fitting procedure described here is independent of the scaling factor used. Following this correction, we compared the single component CO and N_2_ experimental and simulated adsorption performance of HKUST-1 at 283, 298 and 313 K; Fig. 2 shows the adsorption isotherms at these conditions. When applying the scaling factor *ϕ*, the simulations under-predict the experiments significantly because the strength of the Cu–CO and Cu–N_2_ interactions are not fully considered by the DFF. To solve this, we increased the Cu interaction parameter, *ε*, from the Lennard-Jones (LJ) potential, keeping all the other parameters unchanged, until we found agreement over the entire pressure range of the experimental isotherm. We call this new force field DFF+. Table S1† shows the LJ parameters of the original DFF and modified DFF+. Overall, we observed an excellent agreement between experiments and simulations in the range of 283–313 K, with a small underestimation for CO at low pressures. Although we found similar results for N_2_, the increment in ε was substantially smaller (see Table S1†). As mentioned above, this difference is related to the nature of the interaction of CO and N_2_ with the Cu adsorption site. Whereas CO is expected to bind to Cu atoms either through σ-bonds or Cu–CO π-back donation,^15,28^*i.e.* chemisorbed, the smaller quadrupole moment of N_2_ and its zero dipole moment^41^ makes the interaction with the open metal site weaker.

Once DFF+ was adapted to HKUST-1, we tested its transferability on two different MOFs (analogues to HKUST-1). We choose arbitrarily CuTDPAT (CSD reference code, XALXUF), a MOF consisting of Cu–Cu paddle-wheels linked together by TDPAT = 2,4,6-tris(3,5-dicarboxylphenylamino)-1,3,5-triazine linkers and PCN-12 (CSD reference code, HOGLEV), a MOF consisting of Cu–Cu paddle-wheels linked together by 3,3′,5,5′-tetracarboxydiphenylmethane linkers. After synthesizing the materials, we evaluated their performance against our predictions. Fig. S9 and S10† compare the experimental and DFF+ simulated isotherms of CO and N_2_ in CuTDPAT (XALXUF) and PCN-12 (HOGLEV) up to 20 bar and between 283 and 313 K. Taking into account the scaling factor from the N_2_ adsorption isotherms at 77 K (Fig. S7 and S8†) comparison, *ϕ* = 1.05 for CuTDPAT and *ϕ* = 0.92 for PCN-12, DFF+ shows a good match of the experimental data for both CO and N_2_ at all conditions studied, confirming the transferability of the force field, and allowing for fast screening of the 183 identified Cu–Cu paddle-wheel MOF structures.

## 3. High-throughput screening (HTS) and structure–property relationships

To study the MOF subset for separation of CO/N_2_ mixtures, we focus on pressure swing adsorption (PSA), the most widely used industrial process for adsorption-based gas separation. In addition, we included temperature swing adsorption at low temperature (TSA−) and temperature swing adsorption at high temperature (TSA+) processes. In a PSA process, the energy consumption is essentially the “lost work”, whereas in a TSA process it is heat. In both cases, the material is exposed to the gas mixture at adsorption conditions, *i.e.* high pressure for PSA, low temperature for TSA− and a high temperature for TSA+, followed by the regeneration of the material at desorption conditions, by decreasing the pressure or increasing the temperature, depending on the process. In this work, we explored three different conditions: (i) PSA at 298 K, with adsorption at 40 bar and desorption at 1 bar; (ii) TSA− at 1 bar, with adsorption at 200 K and desorption at 298 K; and (iii) TSA+ at 1 bar, with adsorption at 298 K and desorption at 398 K. To evaluate these conditions, we ran multicomponent GCMC simulations on the 183 selected MOFs with a feed gas composition of 50% CO and 50% N_2_. From the adsorption isotherms, we evaluated metrics such as CO uptake and hence working capacity (Δ*N*), using eqn (1),

1 Δ*N*_CO_ = *N*^ads^_CO_ − *N*^des^_CO_

where *N*^ads^_CO_ is the CO uptake and *N*^des^_CO_ is the amount of CO on the adsorbent after completing the desorption step; and CO/N_2_ selectivity, using eqn (2),2
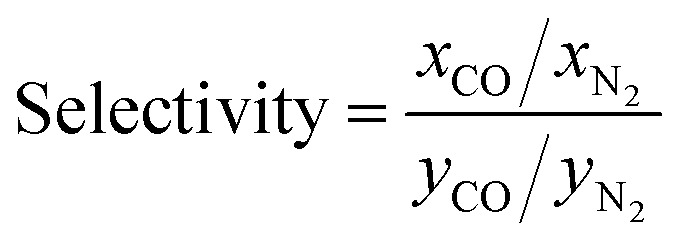
where *x*_CO_ and *x*_N2_ are the mole fractions of CO and N_2_ in the adsorbed phase and *y*_CO_ and *y*_N2_ are the mole fractions of CO and N_2_ in the bulk gas phase; and regenerability – which is a measure of the fraction of adsorption sites that are regenerated during the desorption step – using eqn (3),3
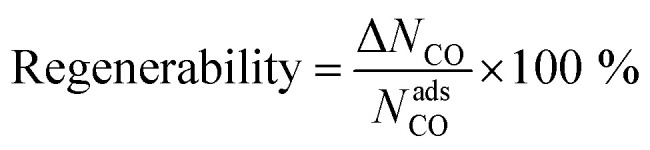
to identify top-performing adsorbents and to establish relationships with structural and geometric-adsorption properties of the MOFs. By being able to understand this landscape of properties, we aim to guide the design and synthesis of new adsorbents with high separation performance.

**Fig. 3:**
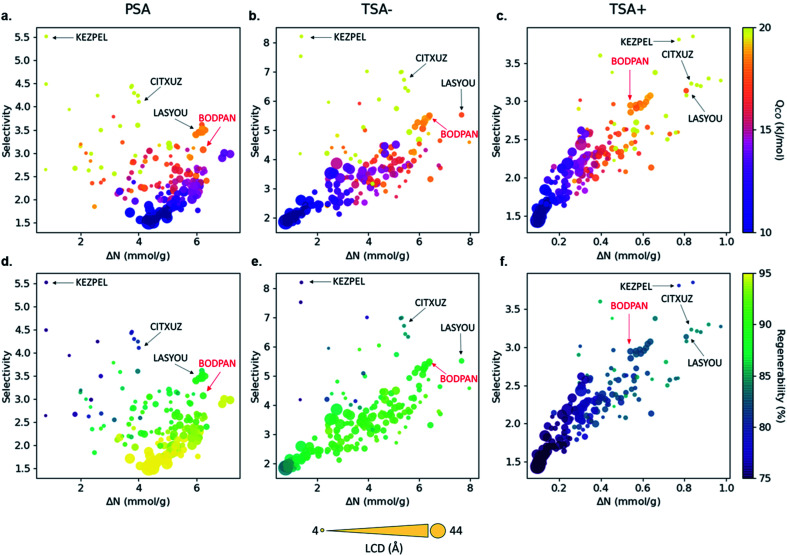
Structure–property relationships obtained from the molecular simulations of 183 MOFs. Selectivity *vs.* working capacity is plotted for PSA (a & d), TSA− (b & e) and TSA+ (c & f) processes, where the color scale represents (a–c) the CO heat of adsorption and (d–f) the regenerability. Symbol size represents the largest cavity diameter (LCD) in Å. Four structures with top performance are named and highlighted, including HKUST-1 (BODPAN), labeled in red. PSA conditions are 298 K, with adsorption at 40 bar and desorption at 1 bar; TSA− conditions are 1 bar, with adsorption at 200 K and desorption at 298 K; TSA+ conditions are at 1 bar, with adsorption at 298 K and desorption at 398 K. The dynamic representation of the properties can be found at https://aam.ceb.cam.ac.uk/mofexplorer.html.

Fig. 3 shows the relationship between CO/N_2_ selectivity, Δ*N*_CO_, isosteric heat of adsorption (*Q*_st_) of CO and regenerability for the 183 MOFs in the three separation processes studied, PSA, TSA− and TSA+. Fig. S11† shows the relationship between selectivity and working capacity with metal density and pore volume. Visualization of the many descriptors in a screening process is just as important as their calculation. For this reason, we have included a website with a dynamic representation (https://aam.ceb.cam.ac.uk/mofexplorer.html), so one can visualize how the different structural descriptors affect the adsorption properties of the selected MOFs included in this study. The overall distribution for each process is very similar, with selectivity increasing when CO *Q*_st_ increases and pore volume decreases. We expected this general trend, given that the higher CO interaction means a higher preference of the MOF towards the molecule, leading to an increase in selectivity. At the same time, high values of *Q*_st_ for CO, ignoring the surface chemistry, are generally related to low pore volumes and narrow porosities. However, the range of values that selectivity reaches for each process is different, with PSA, TSA− and TSA+ showing maximum selectivities of 5.52, 8.21 and 3.85, respectively.

While a high working capacity is desired, a high working capacity alone by no means gives an indication of the material's performance as kinetics becomes important too. A material with a sharp isotherm will give a high working capacity, but the diffusion coefficient for such materials will be extremely low, which means that, in practice, it will take too long to yield the desired working capacities. Similarly, although a high selectivity is desired, it alone would not be sufficient as a material with a high selectivity would adsorb one component so strongly that the energy for regeneration will be really high, impacting its working capacity and making it challenging to use the material in a cyclic fashion. Here, it is important to point out that our study is limited to equilibrium effects.

When looking at the top materials, we identified a trade-off between selectivity and working capacity for the three processes. Among the 183 MOFs with Cu–Cu paddle-wheels studied here, we selected four MOFs as suitable candidates for CO/N_2_ separations based on high selectivity, high working capacity, or a combination of both in PSA and TSA−. We did not apply these criteria to the materials in the TSA+ process as it offers very low working capacities for all the materials. The materials selected are KEZPEL, which offers by far the highest selectivity, but lowest working capacity; CITXUZ, which offers relatively high selectivity and moderate working capacities; LASYOU, which offers slightly lower selectivity but a greater working capacity than CITXUZ; we also included BODPAN (HKUST-1) as a well-known material with comparable performance.

The behavior of the four MOFs in terms of separation is consistent, qualitatively, across the PSA and TSA− processes studied *i.e.* they are showing the highest performance. Although other structures did better than the four MOFs selected, their behavior was not consistent across the two processes, *i.e.* some structures excel in, say, PSA, but perform poorly in TSA− and, hence, did not make it to our list of suitable candidates for CO/N_2_ separations. Another interesting observation is that the CO working capacity depends very strongly on the type of process used for the CO/N_2_ separation, especially for the structures which have an LCD >35 Å. These structures have the highest working capacity in the PSA process but have the lowest working capacity in the TSA− and TSA+ processes. This is the reason why we did not select a MOF with the highest working capacity here – there was not any that performed consistently well across the two processes. The regenerability of MOFs is another property that strongly depends on the type of process used, with regenerability decreasing in the order PSA > TSA− > TSA+. In addition, the MOFs with the highest selectivity also have the highest *Q*_st_ for CO. We also find a strong correlation between the metal density and selectivity, with selectivity increasing with the increase in metal density (Fig. S11†), confirming the hypothesis that the metal sites act, indeed, as critical adsorption sites for the CO molecules.

**Fig. 4:**
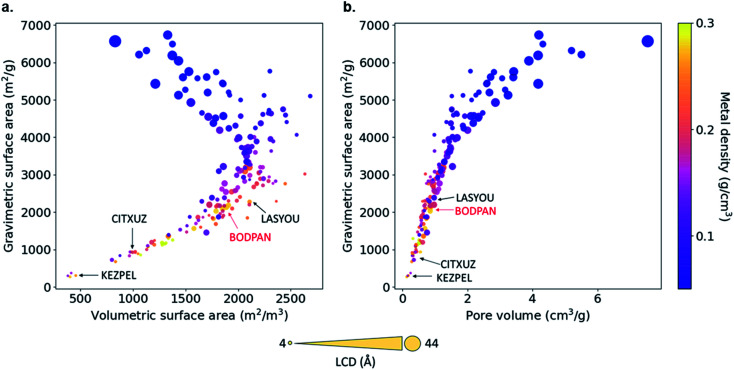
Relationship of textural properties and metal densities of the 183 Cu–Cu paddle-wheel MOFs. (a) Gravimetric *vs.* volumetric surface area, and (b) gravimetric surface area *vs.* pore volume. The size of the symbols indicates the LCD in Å; color scale indicates the MOF metal density. Four structures with top performance are named and highlighted, including HKUST-1 (BODPAN), labeled in red.

Fig. 4 shows the relationship between geometric, textural properties – gravimetric surface area, volumetric surface area, LCD, pore volume – and metal density. The LCD spans from *ca.* 4.56 (ZAWQAR) to 42.80 Å (BAZGAM). The pore limiting diameter (PLD) spans from *ca.* 3.88 Å (ZAWQAR) to 24.25 Å (BAZGAM). Since the kinetic diameters of CO and N_2_ are 3.76 Å and 3.64 Å, respectively, none of these MOFs can actually be used for the size-based separation of CO/N_2_ mixtures. Hence, the only way to actually separate CO from N_2_ would be to adsorb one of the two species selectively on the open metal sites of the MOF. The gravimetric surface area and volumetric surface area range from 269 to 6732 m^2^ g^−1^ and from 381 to 2685 m^2^ cm^−3^. The metal density relates inversely to the LCD, as the Cu–Cu paddle-wheel MOFs with large LCD have extended linkers that diminish the effect of the open-metal sites. All of the analogues are open structures with pore volumes ranging from 0.13 cm^3^ g^−1^ to 7.55 cm^3^ g^−1^.

## 4. Process simulations

Once potentially suitable candidates for CO/N_2_ separations have been identified, it is important to study their performance across a range of process parameters obtained through process simulations. This is required to understand how the properties of a MOF contribute to the overall process performance, and the trade-offs between properties, *e.g.* between selectivity and working capacity. Process simulations are also useful for identifying the most appropriate combination of operating conditions, considering equipment size and cost, operating schedule and cost of heating, cooling and compression, and thus minimizing the capital and operating costs of the separation process.

Simplified adsorption process models provide a means for quick preliminary evaluation of adsorbent performance in adsorption processes. In this work, we have used shortcut process models to evaluate the adsorption process performance. This work uses a three-step shortcut TSA model developed by Ajenifuja *et al.*^42^ and the three-step shortcut PSA model developed by Maring and Webley,^43^ modified by Ajenifuja,^44^ to account for isothermal operation, rather than adiabatic operation, of the PSA adsorption step. Detailed explanations of these simplified process models and procedures for model implementation are summarised in the Methods section and provided in the respective publications.

We used three process performance indicators – purity, cyclic working capacity and recovery – to evaluate adsorbent performance (see eqn (4)–(6)):4
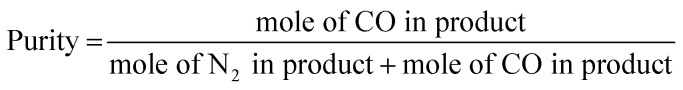

5 Cyclic working capacity = moles of CO produced per unit mass of adsorbent per cycle

6a
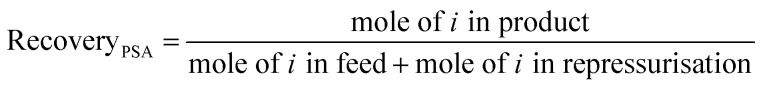
6b
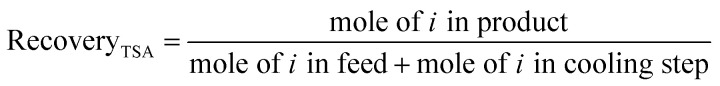
where purity is defined as the mole fraction of CO in the product stream, cyclic working capacity is defined as the amount of CO in the product, and recovery is defined as the fraction of CO recovered after the repressurization/cooling and feed step.

The selection of an appropriate model for binary adsorption isotherms is critical, as the prediction of multicomponent adsorption depends significantly on the selected model. The choice of isotherm has direct implications for adsorption process modelling and the accuracy of subsequent process performance predictions. We used the dual-site Langmuir (DSL) model,^45,46^ which takes into account two distinct, theoretical adsorption sites, such as are found in many MOFs with open metal sites. In the absence of experimental binary adsorption isotherms, predictions from the DSL model have been validated by fitting them to binary GCMC simulated adsorption isotherms (Fig. S13†). Table S4 in the ESI† lists the DSL model parameters.

**Fig. 5:**
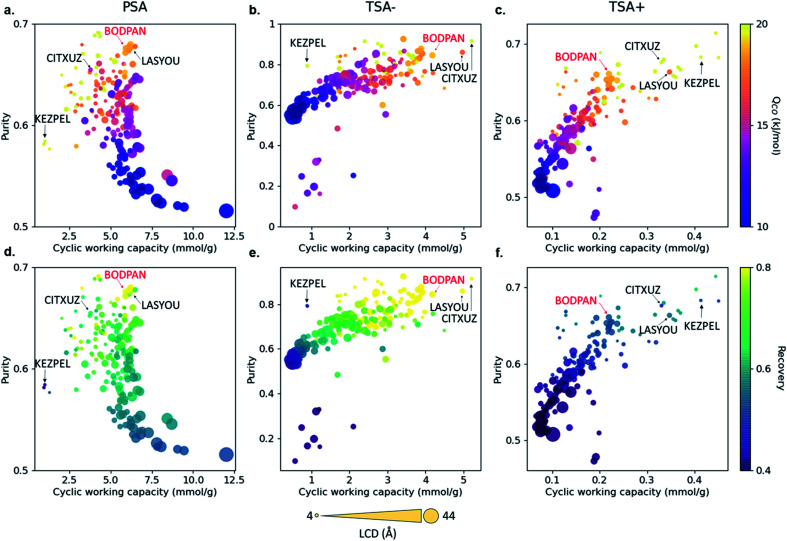
Structure–process relationships obtained from the process simulations of 183 MOFs. Purity *vs.* cyclic working capacity is plotted for PSA, TSA− and TSA+ processes, where the color scale represents (a–c) the CO heat of adsorption and (d–f) the recovery. Symbol size represents the largest cavity diameter (LCD) in Å. Four structures with top performance are named and highlighted, including HKUST-1 (BODPAN), labeled in red. PSA conditions are 298 K, with adsorption at 40 bar and desorption at 1 bar; TSA− conditions are 1 bar, with adsorption at 200 K and desorption at 298 K; TSA+ conditions are 1 bar, with adsorption at 298 K and desorption at 398 K.

Fig. 5 shows the relationship between purity, cyclic working capacity, *Q*_st_ for CO and recovery for the 183 MOFs in the three separation processes studied; Fig. S12† shows the relationship between purity, cyclic working capacity, metal density and pore volume. Purity shows a strong correlation with the *Q*_st_, with purity increasing with the increase in *Q*_st_, whereas the cyclic working capacity does not affect necessarily those MOFs with the highest purity values. Recovery, on the other hand, is heavily process dependent. For example, the recovery for most of the structures in the TSA+ process is low to moderate, whereas the recovery for most of the structures is quite high in the TSA− process, indicating that temperature has a strong influence on the recovery of a material.

Although most, if not all, the materials are better suited in principle to the TSA− process than the PSA one, the latter is by far the more widely used process industrially due to the difficulties in transferring heat to and from commercial-scale TSA beds and the consequent increased cycle times and reduced energy efficiency. Hence, as a case study, we decided to evaluate in more detail the performance of BODPAN, CITXUZ, LASYOU and KEZPEL MOFs across a range of different conditions in the PSA process. To allow for a fair assessment of the different adsorbents, PSA process simulations are performed to investigate the effect of operating parameters on the performance of these adsorbents. Table 1 shows the main results obtained from the molecular and process simulations.

**Table 1:** GCMC and process simulation and adsorption performance indicators of selected MOFs. The performance indicators are calculated for an equimolar mixture of CO and N_2,_ isothermal operation (298 K), desorption at 1 bar and adsorption at 40 bar. Note that the indicators have been obtained from GCMC/Process simulations or experimental data, including powder and monolithic morphologies for HKUST-1 (BODPAN). Volumetric Δ*N* is calculated using single-crystal densities in the GCMC simulated cases and mercury porosimetry in the experimental samples. Surface areas (*S*_a_) are geometrical surface areas in the GCMC simulated cases and BET areas in the experimental ones

Name	Purity (%)	Recovery (%)	Select.	Δ*N* (mmol g^−1^)	Δ*N* (mmol cm^−3^)	Density (g cm^−3^)	*S*_a_ (m^2^ g^−1^)	Regen. (%)
**Calculated using simulated data:**								
BODPAN	67.81	77.63	3.54	6.21	5.53	0.89	2125	89
LASYOU	67.78	67.21	3.61	6.19	5.76	0.93	2275	86
KEZPEL	58.42	43.00	5.52	0.82	1.23	1.50	303	45
CITXUZ	65.71	60.34	4.10	4.01	4.25	1.06	933	79

**Calculated using experimental data:**								
_powd_HKUST-1	57.23	71.73	1.42	6.62	2.85	0.43	1957	83.6
_mono_HKUST-1	69.24	67.99	3.49	4.94	5.34	1.08	1128	85.41

**Fig. 6:**
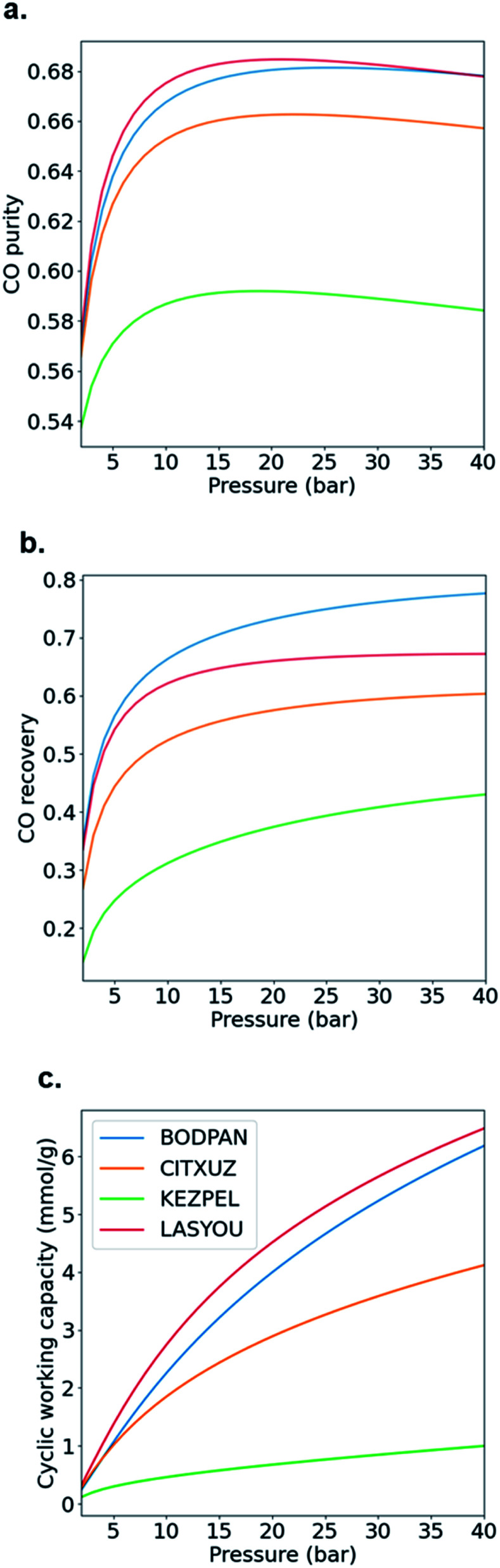
Effect of adsorption pressure on PSA performance. (a) CO purity, (b) CO recovery and (c) CO cyclic working capacity. Desorption pressure is 1 bar and operating temperature is 298 K. Feed consists of an equimolar mixture of CO and N_2_. Blue line, BODPAN (HKUST-1); red line, LASYOU; orange line, CITXUZ; green line, KEZPEL.

Fig. 6 shows how the CO purity, recovery and cyclic working capacity vary with the adsorption pressure (up to a maximum of 40 bar), where the desorption pressure is fixed at 1 bar. This allows us to determine the optimal pressures for each of the adsorbents, *i.e.* the pressure at which the performance is maximized. First, CO purity increases with increasing adsorption pressure, at low pressures, but decreases for higher pressures; these maxima are obtained at 25, 22, 21 and 19 bar for BODPAN (HKUST-1), CITXUZ, LASYOU and KEZPEL, respectively (Fig. 6a). Second, at lower pressures, CO recovery similarly increases with increasing adsorption pressure (Fig. 6b), but at high adsorption pressures (typically >10 bar), increasing the adsorption pressure no longer results in a significant improvement in the amount of CO recovered. Third, the cyclic working capacity of the PSA process increases steadily with adsorption pressure (Fig. 6c). This is expected, as an increase in the adsorption pressure leads to an increase in the working capacity of the adsorbent. KEZPEL has the largest *Q*_st_ for CO and selectivity, and lowest Δ*N*, among these MOFs, and correspondingly exhibits strong adsorption behaviour at low partial pressures. Most CO desorbs during the blowdown step, but less CO desorbs from KEZPEL compared to the other MOFs because of its strong adsorption characteristics. As a result, KEZPEL performs poorly in the PSA process.

**Fig. 7:**
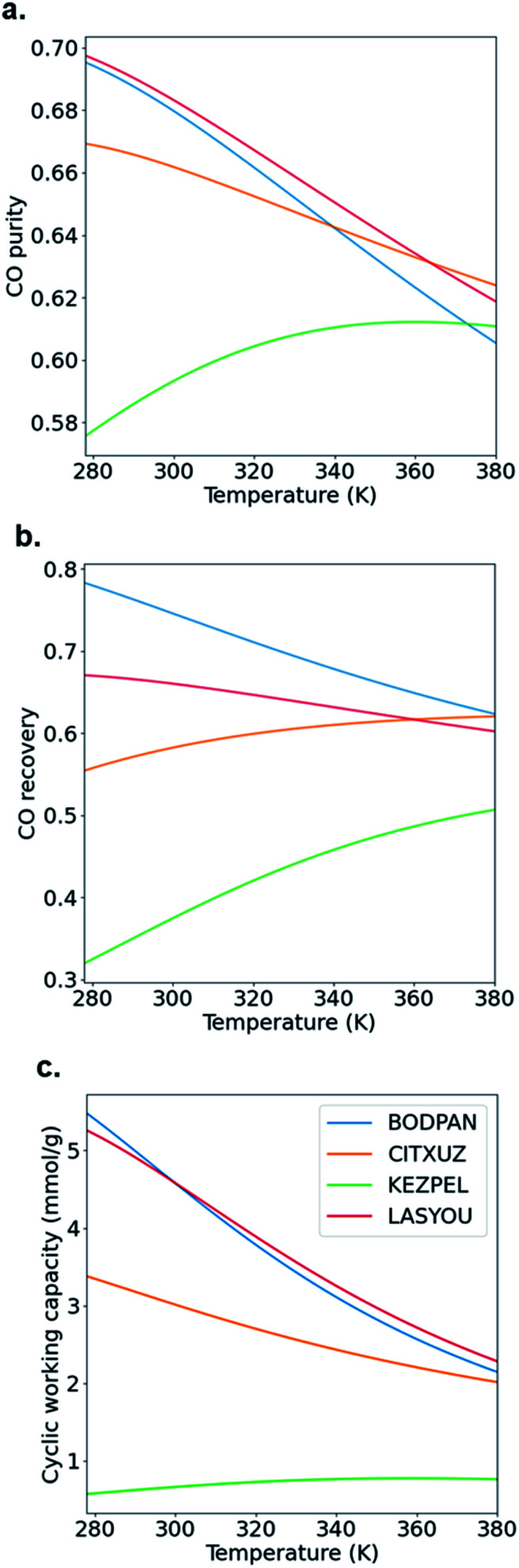
Effect of adsorption temperature on PSA performance. (a) CO purity, (b) CO recovery and (c) CO cyclic working capacity. Desorption pressure is 1 bar and adsorption pressures are 21, 25, 22 and 19 bar for LASYOU, BODPAN, CITXUZ and KEZPEL, respectively. Feed consists of an equimolar mixture of CO and N_2_. Blue line, BODPAN (HKUST-1); red line, LASYOU; orange line, CITXUZ; green line, KEZPEL.

Typical PSA processes are not isothermal because adsorption during the repressurization step is exothermic, whereas the desorption in the blowdown step is endothermic. To evaluate the change in adsorption performance with temperature, we run process simulations over a range of temperatures from 278 K to 378 K. Fig. 7 shows how the CO purity, recovery and cyclic working capacity varies as the adsorption temperature is increased from 273 K to 373 K when studying the MOFs at the optimal pressures obtained from the maximum CO purity (Fig. 6a). The behaviour of BODPAN and LASYOU is quite similar, both quantitatively and qualitatively, in that their performance decreases as the temperature increases. For CITXUZ, the CO purity and cyclic working capacity, both decrease with an increase in temperature, whereas the CO recovery increases with an increase in temperature. For KEZPEL, the CO purity, CO recovery, and cyclic working capacity, all increase with an increase in temperature. Overall, KEZPEL exhibits the lowest CO purity, CO recovery, and CO working capacity; this tells us that a material with a high selectivity alone is not enough, a balance between the working capacity and selectivity must be made. Finally, an important takeaway here is that at low temperatures, the selectivity and working capacity play an important role in determining their process performance; this role gradually diminishes as the temperature is increased – this can be seen by the difference in performance seen at 278 K and 378 K for BODPAN, CITXUZ, and LASYOU. These process simulation results show how a simpler molecular simulation approach, alone, is not enough to screen adsorbents for CO/N_2_ separations. The combined molecular and process simulation approach is necessary for the proper screening of adsorbents, for any application, and gas separation in particular.^23^

Finally, we compare the process and molecular simulation performance indicators of experimentally synthesized materials in our study (Table 1). For this, we used our recent advances in synthesis and engineering of conformed, shaped and densified MOFs into what we call monolithic structures.^47–49^ Importantly, whereas powder HKUST-1 (_powd_HKUST-1) has a low density, the monolithic version (_mono_HKUST-1) shows much higher values. Based on the experimental data obtained for gas adsorption, we found that – on a gravimetric basis – the difference in Δ*N* is negligible, whereas the lower density of _powd_HKUST-1 results in a 48% reduction of the theoretical volumetric Δ*N* of BODPAN. In contrast, the volumetric Δ*N* of _mono_HKUST-1 is only reduced by 3% of the theoretical value, *i.e.* the volumetric Δ*N* of _mono_HKUST-1 is 87% higher than _powd_HKUST-1. We rerun the process simulations based on these two experimental samples to validate the results obtained from molecular simulations. Our approach accurately predicts the performance parameters of HKUST-1 – benchmarked against the process parameters calculated for _powd_HKUST-1 and _mono_HKUST-1 using the experimentally determined adsorption isotherms. All in all, the predicted performance for the densified version of the material, _mono_HKUST-1, is really good, especially when taking into account the fact that the monolith exists in a pelletized form and can withstand the mechanical stresses of operating under a range of different temperatures and pressures as well as the presence of vibrations and the weight of the materials column itself in different industrial settings. This is in contrast with other traditional pelletization methods that tend to collapse the porosity or achieve very low densities when applying pressure.

## 5. Outlook

We presented here a generic toolbox approach combining molecular and process simulations, data visualization, experimental synthesis and characterization, and testing of materials. As a case study, we take the challenging CO/N_2_ separation – two molecules with very similar sizes and physical properties – and focus on the recovery of CO as it is a key feedstock of the CATIVA™ acetic acid production process. We show that by combining the molecular- and process-level simulations with the experimental synthesis and characterization approaches, we can accurately and quickly screen a database of materials to identify the optimal ones for a particular application. In this sense, while most studies generally focus on screening materials based on molecular simulations only, this work extends this approach to consider a range of process operating modes (PSA, and above- and below-ambient TSA− and TSA+ processes), together with process simulations predicting the performance of the overall process, exploring a range of operating conditions. This approach allows us to better understand which material may be the best performing one based on a set of performance indicators determined *via* simplified process simulations (*i.e.* product purity, component recovery, and cyclic working capacity of the process), and informed by knowledge of more fundamental properties predicted by molecular simulations (*i.e.* selectivity and working capacity).

To run the molecular simulations, we first adapted the DREIDING force field on HKUST-1 to reproduce the stronger interaction of the Cu–Cu paddle-wheel adsorption sites with CO and N_2_. We then tested the transferability of this force field on two daughter Cu–Cu paddle-wheel structures (CuTDPAT; CSD identifier, XALXUF and PCN-12; CSD identifier, HOGLEV) and validated the predicted adsorption isotherms by comparison to measured experimental curves. With the adapted force field, we ran molecular simulations on the 183 MOFs included in the CSD MOF subset, analyzing the role of the different textural properties and metal density on their adsorption descriptors. We also carried out process simulations applying these 183 MOFs for the three adsorption processes (PSA, TSA− and TSA+) to further analyze their performance. The process simulations enabled preliminary comparison of the performance of these adsorption processes, in terms of the purity of the desired product, the recovery of the species of interest and the amount of product generated per unit mass of adsorbent.

Four MOFs were selected for more detailed evaluation – one with high selectivity (KEZPEL), one with high working capacity (LASYOU), one with good selectivity and good working capacity (CITXUZ), and HKUST-1 (also referred to as BODPAN, providing a performance benchmark). The study then focused on the application of these four materials for PSA, the most established operating mode for adsorption-based gas separation processes. These process-level simulations predicted how these materials would perform across a wide range of industrially relevant operating conditions (pressure and temperature). These PSA process simulations revealed that (i) one should give equal importance to the selectivity and working capacity when selecting a suitable material for CO/N_2_ separations, and (ii) at low temperatures, the selectivity and working capacity play an important role in the process performance; this role diminishes at higher temperatures. Interestingly, HKUST-1 (BODPAN) was one of the first reported MOFs but, still, twenty years later, is one of the top-performing materials in numerous adsorption applications. Obviously, HKUST-1 presents various challenges for industrial application not considered here, such as the lack of long-term stability when exposed to moisture, which could prevent its industrial use.

As a result of the selection process, we used our recent advances in the densification of MOFs to synthesize an HKUST-1 monolith and validated our whole molecular-process simulation results with experiments. The monolithic form of the MOF, _mono_HKUST-1, is determined to be, to the best of our knowledge, one of the best materials, at the conditions studied, for the separation of CO/N_2_ mixtures industrially due to its high density, surface area, mechanical durability and good performance characteristics in the PSA process.

## 6. Methods

### 6.1 Molecular simulation details

N_2_ and CO adsorption was simulated using the grand canonical Monte Carlo (GCMC) method as implemented in the RASPA molecular simulation package.^50^ A total of 183 MOFs having Cu–Cu paddle-wheels were selected from the CORE MOF database, a subset of the Cambridge Structural Database (CSD). It is important to note that the residual solvents present in the selected structures were removed using a previously developed code.^7^ We used atomistic models, in which the framework atoms are kept fixed at their crystallographic positions. Partial charges to the framework atoms were assigned using the EQeq protocol.^51^ Lennard Jones (LJ) and electrostatic potentials are used to model the interatomic interactions between the framework atoms and gases. LJ potential parameters for the framework atoms are taken from the DREIDING force field (DFF).^31^ The LJ parameters for Cu have not been described in the DREIDING force field, and are taken from the Universal Force Field (UFF).^32^ N_2_ is described using LJ parameters taken from the TraPPE force field^52^ and a 3-site model proposed by Martin-Calvo *et al.*^29^ is used to describe CO (Table S2†). Lorentz–Berthelot mixing rules are used for all the cross-interaction terms. All the long-range electrostatic interactions are calculated using the Ewald summation method. A cutoff radius of 12.8 Å is applied to the LJ interactions. Periodic boundary conditions are applied in all three dimensions. Peng–Robinson equation of state is used to convert the pressure to the corresponding fugacity used in the GCMC simulations. For all the pressure points we use 20 000 cycles for equilibration and another 20 000 cycles to average the properties. A cycle is defined as the maximum of 20 steps or the number of molecules in the system; this implies that on average, a Monte Carlo move has been attempted on all the molecules during each cycle. Monte Carlo moves consist of insertion, deletion, translation and rotation moves with equal probabilities. Geometric properties for all the frameworks in the database are computed using the Zeo ++ simulation package, details of which are given in the ESI.†

### 6.2 Process simulation

Simplified PSA and TSA process models developed by Maring and Webley^43^ and Ajenifuja *et al.*^42,44^ were used for process-level adsorbent screening. The PSA model features a cycle with adsorption, blowdown, and repressurization steps. The TSA model features a cycle with adsorption, heating, and cooling steps. The simplified models assume that instantaneous equilibrium is achieved between the gas and solid phases so that the mass balances given below apply at all times in the cycle. Isothermal adsorption is assumed in both the PSA and TSA cycles, rather than an adiabatic adsorption step, as is assumed in the PSA model developed by Maring and Webley.^43^ The models also assume that the specific heat capacity of the gas phase is negligible compared to the heat capacity of the solid adsorbent. Another important assumption is that of neglecting the effect of the column wall on the heat transfer to and from the bed. This assumption substantially reduces the complexity of the energy balance.

The total moles of species *i* and *j* in the system at any time are calculated from:7
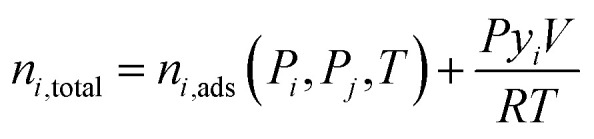
8
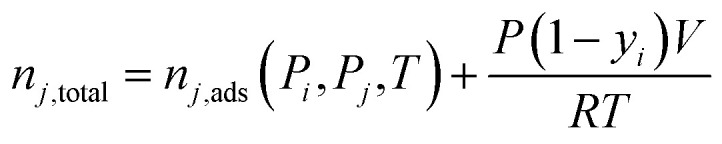
where *P* is pressure, *y*_*i*_ is the mole fraction of species *i*, *V* is the total void volume, *R* is the universal gas constant and *T* is temperature. *n*_*i*,ads_ and *n*_*j*,ads_ are the uptake of species *i* and *j* as described by equilibrium isotherm models.

The temperature change in the bed can be calculated from the following simplified energy balance:

9 *m*_ads_*C*_p_Δ*T* = *Q*_i_Δ*n*_*i*,ads_ + *Q*_*j*_Δ*n*_*j*,ads_ + *Q*_ext_

where *m*_ads_ is the mass of adsorbent, *C*_p_ is the heat capacity of adsorbent, *Q*_*i*_ and *Q*_*j*_ are the isosteric heats of adsorption of species *i* and *j*, and *Q*_ext_ is the amount of heat provided by an external source.

The isosteric heats of adsorption are calculated using the Clausius–Clapeyron equation:10
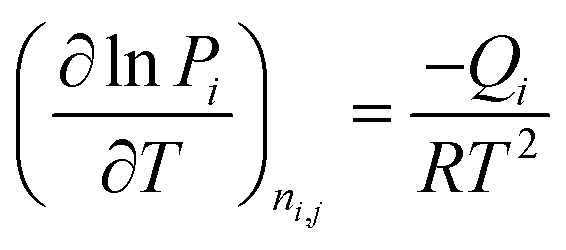


The unit volume of the bed is calculated per unit mass of adsorbent, the specific void volume, *ε*_total_, and the bed density, *ρ*_bed_:11
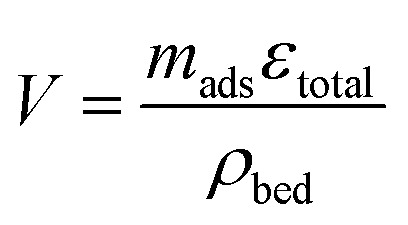


The model is implemented in MATLAB and solved to predict process performance given isotherm parameters, adsorbent physical properties, feed conditions and feed composition. The results are expressed per kg of adsorbent per cycle. Further details about the implementation of the models are available in the respective original publications.^42–44^

The dual-site Langmuir isotherm is used to predict adsorption equilibria. The single-component DSL isotherm equation is:12
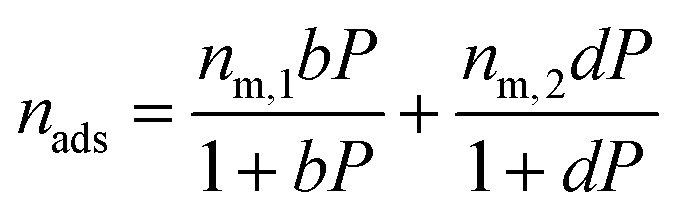
where *n*_m_ is the monolayer saturation capacity at each adsorption site; *b* and *d* are the affinity parameters for each adsorption site.

The binary mixture DSL isotherm equations are:13
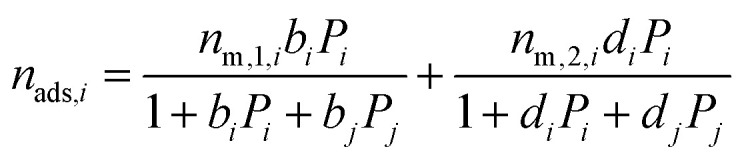
14
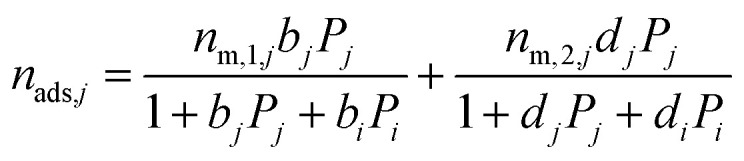


The temperature dependence of the affinity parameters (*b* and *d*) can be incorporated in the model using Arrhenius-type equations. For any component, *i*:15
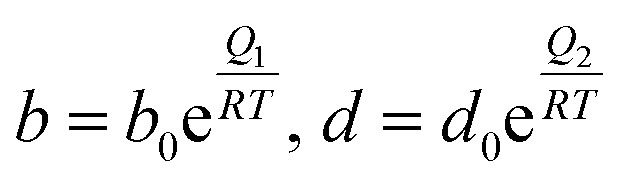
where *b*_0_ and *d*_0_ are the affinity constants for each adsorption site at reference conditions.

### 6.3 Experimental details

#### General considerations

Reagents were obtained from commercial sources and used as received unless otherwise noted.

### Synthesis of _powd_HKUST-1 (BODPAN)

HKUST-1 was synthesised using a method adapted from the literature.^53^ A 12 ml solution of dimethylformamide (DMF)/ethanol (EtOH)/H_2_O (1 : 1 : 1 v/v) was used to dissolve 1,3,5-benzenetricarboxylic acid (H_3_BTC, 0.901 g), this was added to a 12 ml solution of DMF/EtOH/H_2_O (1 : 1 : 1 v/v) with Cu(NO_3_)_2_·2.5H_2_O (1.321 g) to provide a blue solution. This solution was added to a 100 ml Schott bottle and placed in an 80 °C oven for 16 h. The solution was allowed to cool and was filtered and washed with DMF and EtOH to yield a blue powder.

### Synthesis of _mono_HKUST-1 (BODPAN)

_mono_HKUST-1 was synthesized based on a modification of the synthesis method of HKUST-1 reported by Wee and colleagues.^54^ Solutions of BTC (10 ml, 0.062 M) and Cu(NO_3_)_2_·2.5H_2_O (10 ml, 0.064 M) in ethanol were mixed and stirred for 10 min at room temperature (20 ± 1 °C). After centrifugation, the solid was kept in the Falcon tube and washed in ethanol for 10 min (15 ml, 3 times) and then dried in an incubator at room temperature (20 ± 1 °C) overnight. The solid was then transferred to a glass vial and was further dried at 120 °C in an incubator under vacuum overnight.

### Synthesis of Na_6_TDPAT

Na_6_TDPAT (5,5′,5′′-(s-triazine-2,4,6-triyltriimio)triisophthalic acid hexasodium salt) was prepared according to the literature procedure.^55^ 5-Aminoisophthalic acid (4.514 g), H_2_O (50 ml) and NaOH (2 M, 20 ml) were added to a 250 ml round bottom flask. NaHCO_3_ (2.243 g) was added and the solution was stirred whilst being cooled to 0 °C. A solution of cyanuric chloride (1.142 g) and acetone (7 ml) was prepared and added dropwise to the round bottom flask. The solution was refluxed for 24 h at 105 °C and then cooled to room temperature and filtered. The white filtrate was washed with EtOH/H_2_O (3 : 1 v/v), EtOH, and Et_2_O. The solid was dried overnight in a 70 °C oven. The solid was added to H_2_O (60 ml) and the solution's pH was increased to 9 using NaOH (2 M). The solution was filtrated and EtOH (75 ml) was added to the filtrate resulting in a white precipitate. This solid was collected by filtration and washed with EtOH and Et_2_O to provide the final product.

### Synthesis of CuTDPAT (XALXUF)

CuTDPAT was synthesised using an adapted method from the literature.^55^ A solution of Na_6_TDPAT (0.901 g), Cu(NO_3_)_2_·2.5H_2_O (1.03 g), DMF (100 ml) and HNO_3_ (30 ml, 3.5 M) was added to a 1 L Schott bottle. The mixture was stirred until no solid remained. The blue solution was placed within a 65 °C oven for 7 days. The solution was allowed to cool to room temperature and a teal powder was removed *via* filtration. The powder was washed with DMF and MeOH to provide the final product.

### Synthesis of PCN-12 (HOGLEV)

PCN-12 was synthesised using a method adapted from the literature.^56^ A solution of 5,5′-methylenediisophthalic acid (H_4_MDIP, 100 mg), Cu(NO_3_)_2_·2.5H_2_O (250 mg) and *N*,*N*-dimethylacetamide (DMA, 15 ml) were sonicated until dissolved. The solution was added to a 20 ml scintillation vial and was heated at 85 °C for 48 h. The solution was allowed to cool to room temperature and the blue precipitate was washed with DMA over 2 days. The blue precipitate was washed further with methanol and dichloromethane (DCM) to provide the final product.

Initial phase characterisation of all materials was achieved using PXRD. Fig. S1–S4† show phase pure materials when compared to their predicted patterns.

N_2_ adsorption isotherms were measured at 77 K. Prior to activation, all materials were solvent exchanged with methanol over three days at room temperature to remove the remaining DMF. Samples were activated at 100 °C. Fig. S5† shows the adsorption isotherms of N_2_ at 77 K in _powd_HKUST-1, CuTDPAT, PCN-12 and _mono_HKUST-1. Samples for high-pressure adsorption isotherms were activated under the same conditions as the 77 K isotherms. Isotherms of both pure CO and N_2_ were recorded at 283, 298 and 313 K and are shown in Fig. S9–S10† and 2. Mercury porosimetry was obtained up to a final pressure of 2000 bar using an AutoPore IV 9500 instrument from Micromeritics. Prior to the analysis, all samples were activated overnight at 120 °C (vacuum) and then degassed *in situ* thoroughly before the mercury porosimetry. We estimated the bulk density of _mono_HKUST-1 at atmospheric pressure.

## Data availability

The data is available through the visualization tool, which can be found here: https://aam.ceb.cam.ac.uk/mofexplorer.html.

## Author contributions

D. F. J. designed the research. M. S. C. and A. E. performed the material synthesis and characterization under the supervision of C. P. and M. P. A., respectively. N. R., A. T. and C. B. did the molecular simulations under the supervision of R. B. P., P. Z. M. and D. F.-J., while A. A. did the process simulations under the supervision of M. J. Technical guidance was provided by D. J. L. and L. W. B. N. R. and D. F. J. wrote the first draft of the manuscript with input from all the authors. All the authors contributed to the final version.

## Conflicts of interest

D. F.-J. has a financial interest in the start-up company Immaterial, which is seeking to commercialize metal–organic frameworks. The remaining authors declare no competing interests.

## Supplementary Material

SC-012-D1SC01588E-s001
