# Supplementary material for: The development of a comprehensive toolbox based on multi-level, high-throughput screening of MOFs for CO/N2 separations
Source: Chem Sci. 2021 Aug 11;12(36):12068–81. doi: 10.1039/d1sc01588e (PMC8457378; doi:10.1039/d1sc01588e)
Supplement: SC-012-D1SC01588E-s001 [file SC-012-D1SC01588E-s001.pdf]

Electronic Supplementary Material (ESI) for Chemical Science.
This journal is © The Royal Society of Chemistry 2021

Supporting information for:

The development of a comprehensive toolbox based on multi-level, high-throughput screening of MOFs for CO/N_2 separations

Nakul Rampal,[a,‡] Abdulmalik Ajenifuja,[b,‡] Andi Tao,[a,‡] Christopher Balzer,[a] Matthew Cummings,[c] Arwyn Evans,[d] Rocio Bueno-Perez,[a] David J. Law,[e] Leslie W. Bolton,[f] Camille Petit,[d] Flor Siperstein,[b] Martin P. Attfield,[c] Megan Jobson,[b] Peyman Z. Moghadam[a,g] and David Fairen-Jimenez[a,*]

[a]*The Adsorption & Advanced Materials Laboratory (A^{2}ML), Department of Chemical Engineering & Biotechnology, University of Cambridge, Philippa Fawcett Drive, Cambridge CB3 0AS, UK.*

[b]*Department of Chemical Engineering and Analytical Science, The University of Manchester, Oxford Road, Manchester M13 9PL, UK*

[c]*Centre for Nanoporous Materials, Department of Chemistry, The University of Manchester, Oxford Road, Manchester M13 9PL, UK*

[d] *Barrer Centre, Department of Chemical Engineering, Imperial College London, South Kensington Campus, London SW7 2AZ, UK*

[e]*bp Chemicals Limited, Saltend, Hull HU12 8DS, UK*

[f]*bp International Limited, Chertsey Road, Sunbury-upon-Thames TW16 7BP United Kingdom*

[g]*Department of Chemical and Biological Engineering, University of Sheffield, Sheffield S1 3JD, UK*

[*]*E-mail: df334@cam.ac.uk*

[‡]These authors contributed equally

Table of contents:

S1. Molecular simulation details 2

S2. Powder X-ray diffraction patterns 4

S3. N_2 adsorption isotherms 6

S4. Force field modification 7

S5. Performance correlations with other commonly used properties 10

S6. Isotherm fitting 12

S7. References 14

S1. Molecular simulation details

Table S1 | Mixed Lennard-Jones parameters of DFF and DFF+ Cu and N_2/CO after applying Lorentz-Berthelot mixing rules. Columns 1 and 2 list the atom types of the pair of atoms for which the mixed Lennard-Jones parameters have been calculated using (i) DFF in Columns 3 and 4, and (ii) DFF+ in Columns 5 and 6. σ is in Å, ε/K_b is in K. ε_{ij} of Cu was scaled by a factor of 23 for CO, and 12 for N_2 in order to achieve a good fit over the whole pressure range of the isotherm.

Atom Type 1	Atom Type 2	DFF		DFF+	
		**ε/k$_B$ (K)**	**σ (Å)**	**ε /k$_B$ (K)**	**σ (Å)**
Cu	C	6.38	3.38	146.63	3.38
Cu	O	15.71	3.05	361.33	3.05
Cu	M_{CO}	0.00	0.00	0.00	0.00
Cu	N	9.52	3.21	114.25	3.21
Cu	M_{N2}	0.00	0.00	0.00	0.00

Table S2 | Lennard Jones parameters and charges for CO and N_2. Column 1 lists the name of the adsorbate; column 2 lists the corresponding atom types of the adsorbate, M_{CO} is the dummy atom of CO and M_{N2} is the dummy atom of N_2; columns 3, 4 and 5 list their corresponding LJ parameters and charges, σ in Å, ε/K_b in K, and q in e taken from the work of Martin-Calvo et al. for CO [1] and from the TraPPE force field for N_2 [2].

Adsorbate	Atom	LJ Parameters		q (e)
		**σ (Å)**	**ε/k$_B$ (K)**	
CO	C	3.636	16.141	-0.2424
	O	2.979	98.014	-0.2744
	M_{CO}	0	0	0.5168
N_2	N	3.31	36	-0.482
	M_{N2}	0	0	0.964
	N	3.31	36	-0.482

Table S3 | Lennard Jones parameters for the atoms in the framework. Column 1 lists the atom type; columns 2 and 3 list the Lennard Jones parameters for the corresponding atom types, σ in Å and ε/K_b in K, taken from the DREIDING force field. . For Cu, which was not available in the DREIDING force field, UFF parameters are used.

Atom	LJ Parameters	
	**σ (Å)**	**ε/k$_B$ (K)**
C	3.470	47.854
H	2.844	7.650
N	3.260	38.948
O	3.030	48.156
F	3.090	36.482
B	3.581	47.838
S	3.590	173.223
Br	3.519	186.316
Cu	3.472	2.518
Zn	4.540	27.676

Absolute adsorption data reported by GCMC was used to compute the excess amounts that can be directly compared with experimental data using the equation (1):

$$N_{total} = N_{excess} + \rho_{gas} V_p \qquad [1]$$

where ρ_{gas} is the bulk density of the gas at simulation conditions, and V_p is the pore volume and equivalent to helium void fraction calculated by the Widom insertion method [3-5]. In this numerical Monte Carlo integration technique, helium was modelled as an LJ fluid (σ = 2.58 Å, ε/k_B = 10.22 K) and the force fields used for the framework atoms were the same as those used in the GCMC simulations. The isosteric heat of adsorption (Q_{st}) was also calculated by the Widom insertion method.

Calculation of geometric properties

We use Zeo++ to characterize each MOF structure. Zeo++ uses Voronoi decomposition to identify probe-accessible regions of void space and calculate the accessible surface area, accessible volume, largest cavity diameter (LCD), and pore limiting diameter (PLD) [6]. Geometric surface area calculations are performed using a probe of radius 1.86 Å, corresponding to N_2. Pore volume calculations use a probe of radius set to zero. Covalent radii from the CSD are used for all MOF atoms.

S2. Powder X-ray diffraction patterns

A Cu source (λ=1.5418 Å) X'pert powder X-ray diffractometer (Malvern Panalytical, UK) was used in a Bragg-Brentano orientation. Samples were manually ground and deposited onto a zero background plate. Scans were recorded from 5-50 º 2θ for a duration of 25 minutes.

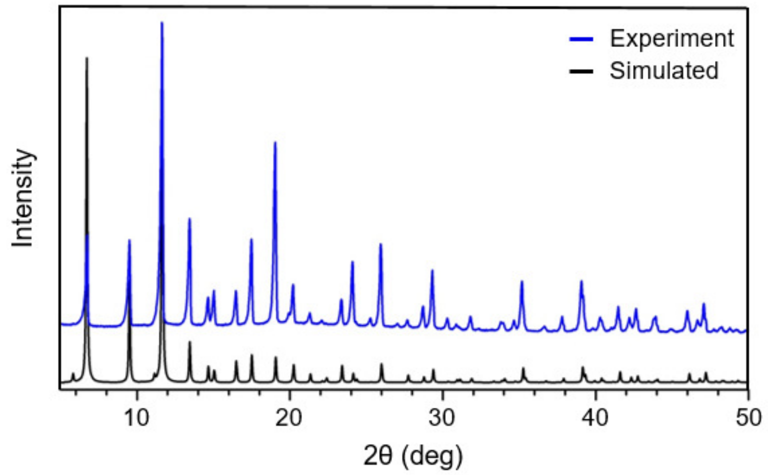

Figure S1 | PXRD pattern of $_{powd}$HKUST-1 (Blue is the experimental pattern, black is the simulated pattern derived from the single crystal structure).

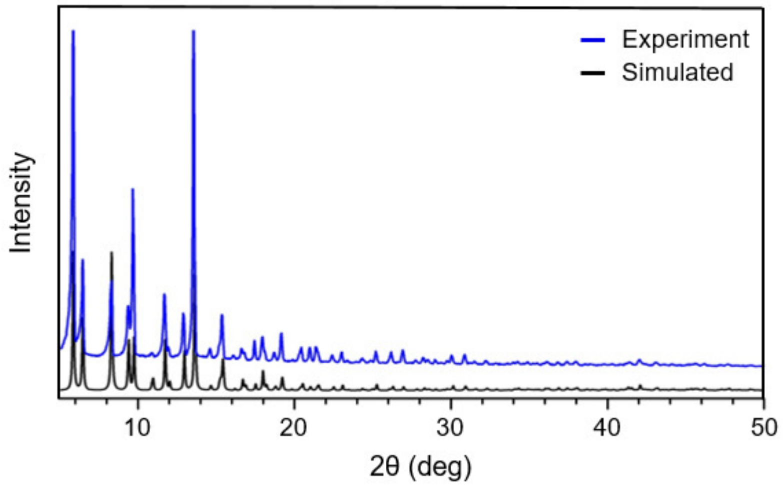

Figure S2 | PXRD pattern of CuTDPAT (Blue is the experimental pattern, black is the simulated pattern derived from the single crystal structure).

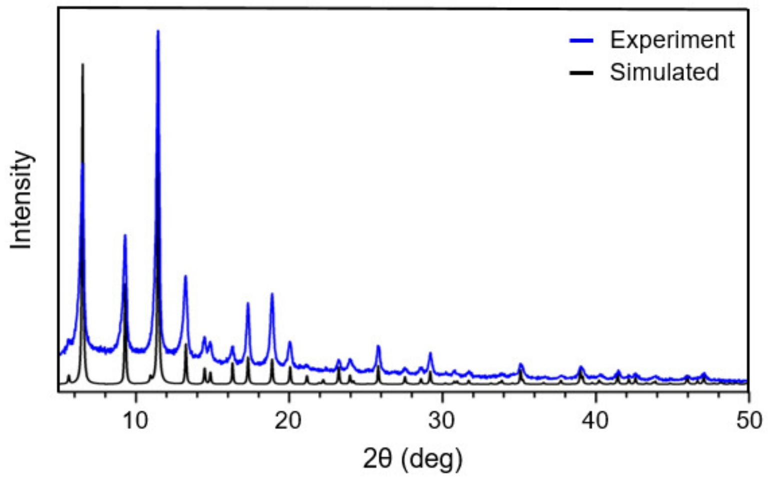

Figure S3 | PXRD pattern of $_{mono}$HKUST-1 (Blue is the experimental pattern, black is the simulated pattern derived from the single crystal structure).

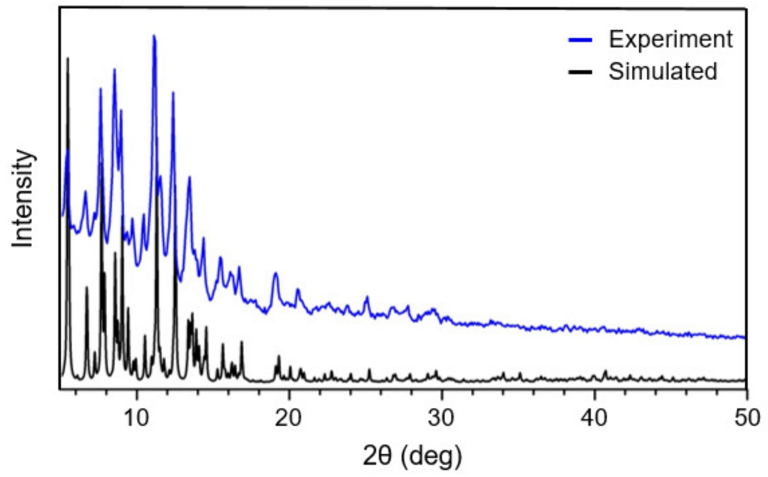

Figure S4 | PXRD pattern of PCN-12 (Blue is the experimental pattern, black is the simulated pattern derived from the single crystal structure).

S3. N_2 adsorption isotherms

Volumetric N_2 adsorption isotherms at 77 K were collected using an ASAP-2020 (Micromeritics, US). Samples, ~70 mg, were loaded into a preweighed volumetric tube and activated at 100 °C. The dry sample mass was then calculated and then transferred to the analytical stage. The free space of the tube was calculated using He and corrected for and the N_2 sorption isotherm was recorded (**Figure S5**). Ultrapure gases were supplied by BOC and temperature control was maintained by a liquid N_2 bath. The BET [7] areas were calculated using the software provided by Micromeritics.

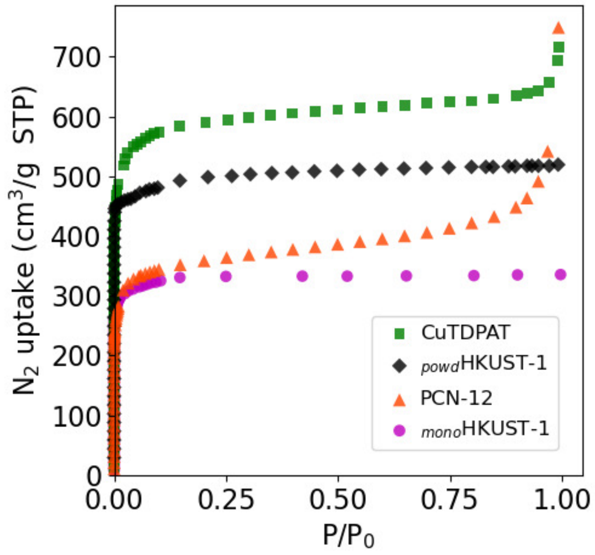

Figure S5 | N_2 adsorption isotherms at 77 K. $_{powd}$HKUST-1 in black (diamond), CuTDPAT in green (squares), PCN-12 in orange (triangles) and $_{mono}$HKUST-1 in magenta (circles) obtained experimentally.

Gravimetric adsorption isotherms

Single component isotherms of both CO and N_2 were achieved by using an Intelligent Gravimetric Analyser (IGA-001, Hiden Isochema, UK) from 0-20 bar. In general, ~70 mg of sample was loaded into the sample chamber and activated at 100 °C at high vacuum. High purity gases, supplied by BOC, were used and the weight was recorded at set pressure points once equilibrium had been reached. To offset the contribution from the buoyancy of the materials, the skeletal density of each material was calculated from a 298 K He isotherm.

S4. Force field modification

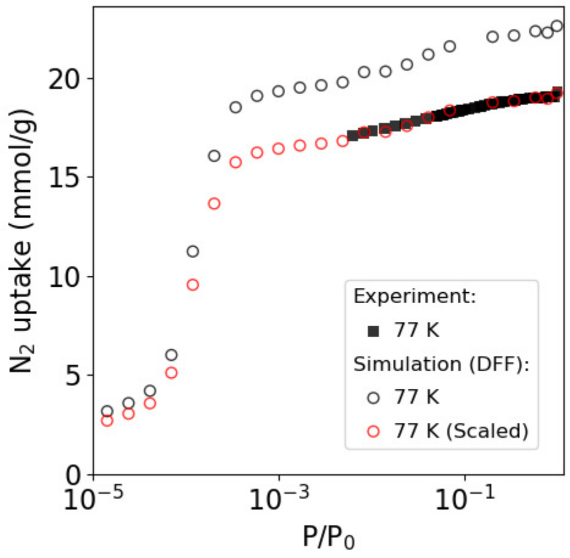

Figure S6 | N_2 adsorption isotherms of $_{powd}$HKUST-1 at 77 K. Filled squares represent the experimental isotherm; the black circles represent the simulated adsorption isotherm; the red circles represent the simulated adsorption isotherms scaled by a factor of 0.85 for $_{powd}$HKUST-1.

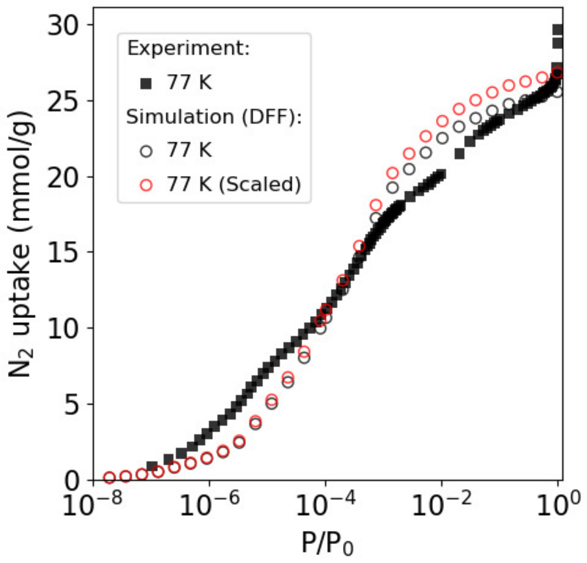

Figure S7 | N_2 adsorption isotherms of CuTDPAT at 77 K. Filled squares represent the experimental isotherm; the black circles represent the simulated adsorption isotherm; the red circles represent the simulated adsorption isotherms scaled by a factor of 1.05 for CuTDPAT.

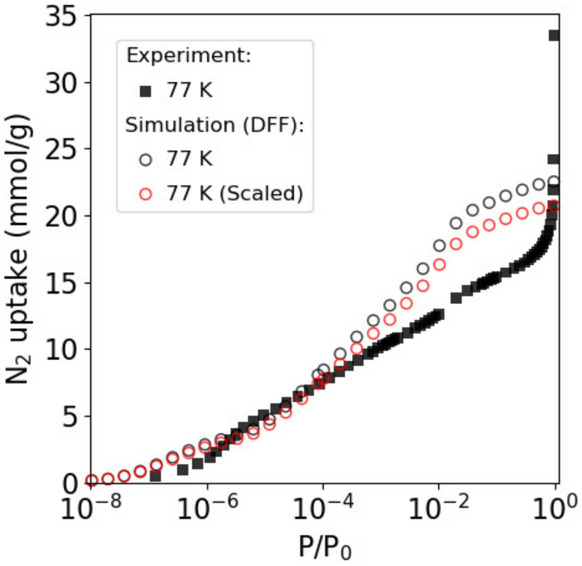

Figure S8 | N_2 adsorption isotherms of PCN-12 at 77 K. Filled squares represent the experimental isotherm; the black circles represent the simulated adsorption isotherm; the red circles represent the simulated adsorption isotherms scaled by a factor of 0.92 for PCN-12.

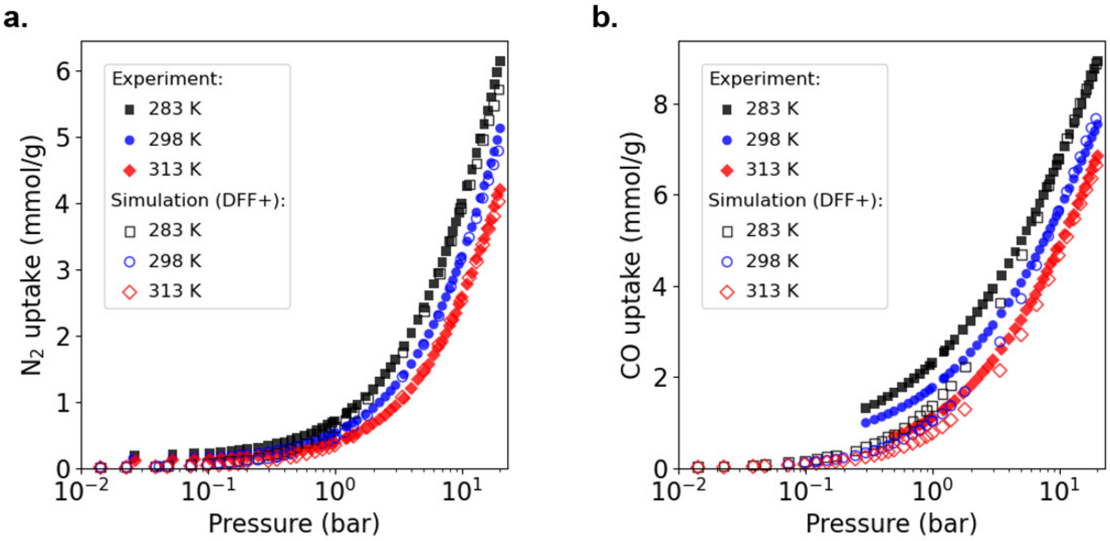

Figure S9 | Experimental and GCMC adsorption isotherms for N_2 (left) and CO (right) in CuTDPAT at different temperatures. Experimental isotherms are shown using filled points and simulated isotherms are shown using hollow points. Different colours and shapes represent the different temperatures; 313 K - red (diamond); 298 K – blue (circles); and 283 K – black (squares).

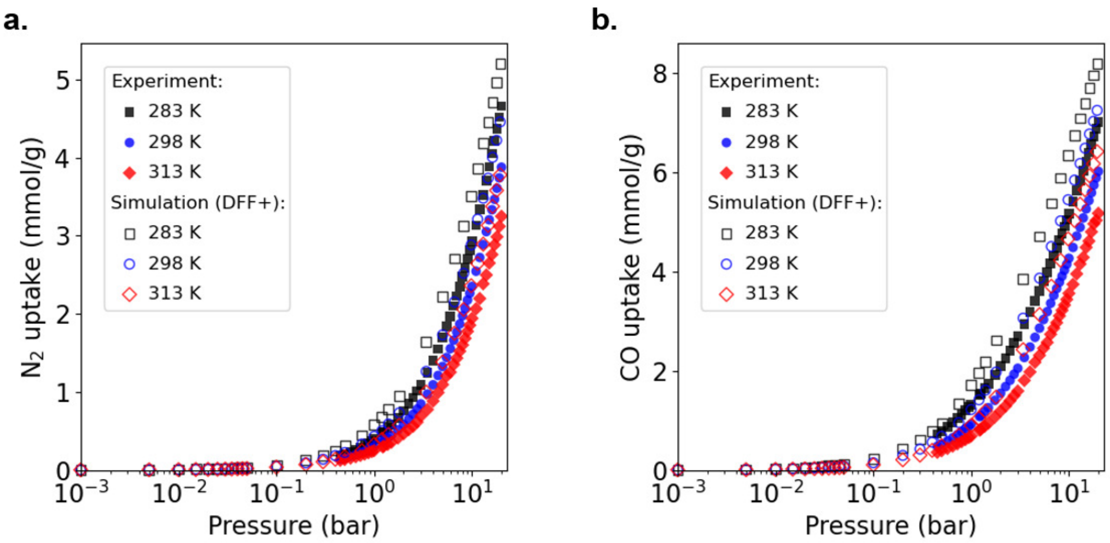

Figure S10 | Experimental and GCMC adsorption isotherms for N_2 (left) and CO (right) in PCN-12 at different temperatures. Experimental isotherms are shown using filled points and simulated isotherms are shown using hollow points. Different colors and shapes represent the different temperatures; 313 K - red (diamond); 298 K – blue (circles); and 283 K – black (squares).

S5. Performance correlations with other commonly used properties

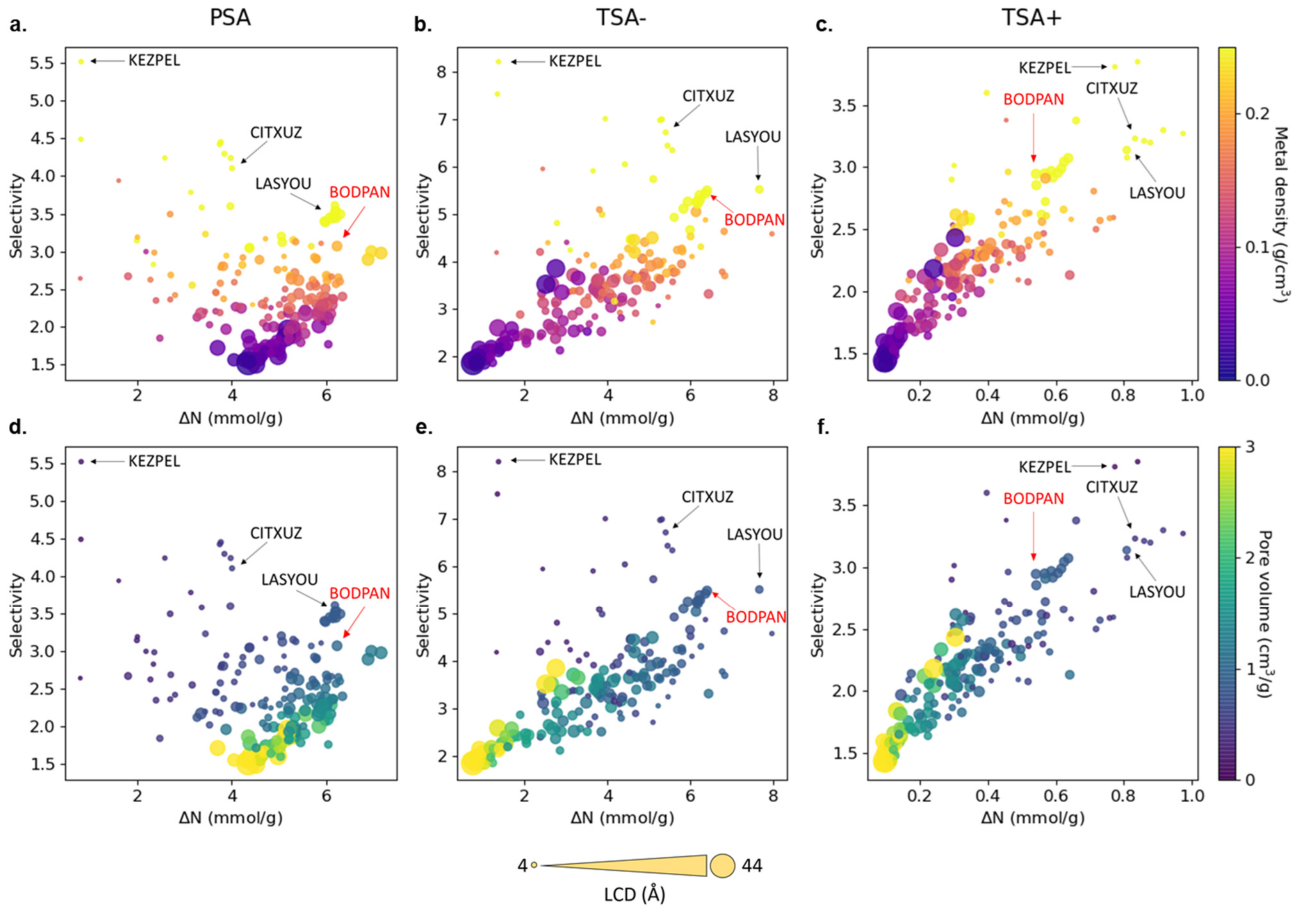

Figure S11 | Structure-property relationships obtained from the molecular simulations of 183 MOFs. Selectivity vs. working capacity is plotted for PSA, TSA– and TSA+ processes, where the color scale represents **a-c** the metal density (g/cm^3) and **d-f** the pore volume (cm^3/g). Symbol size represents the largest cavity diameter (LCD) in Å. Four structures with top performance are named and highlighted, including HKUST-1 (BODPAN), labeled in red. PSA conditions are 298 K, with adsorption at 40 bar and desorption at 1 bar; TSA– conditions are 1 bar, with adsorption at 200 K and desorption at 298 K; TSA+ conditions are at 1 bar, with adsorption at 298 K and desorption at 398 K.

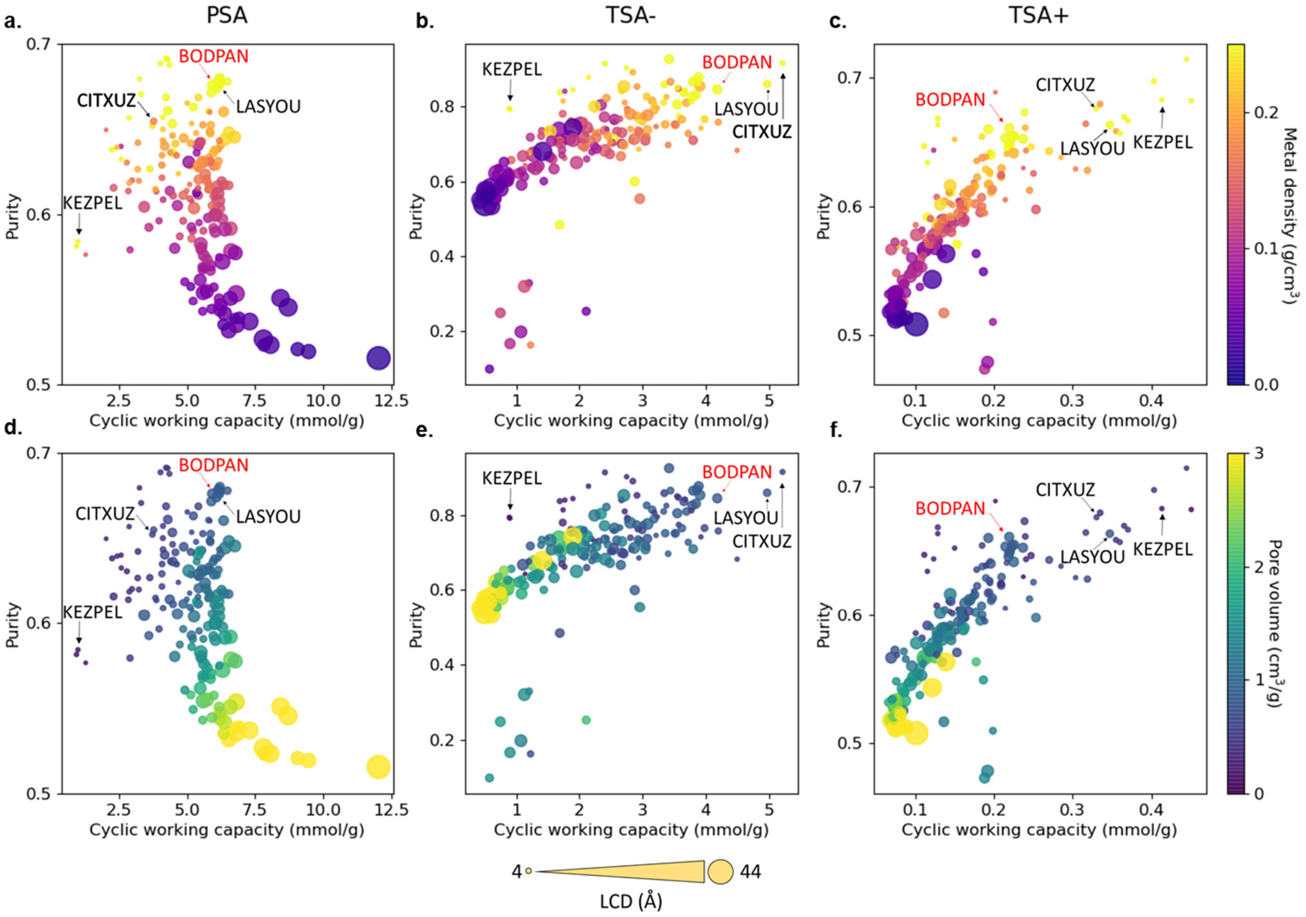

Figure S12 | Structure-process performance relationships obtained from the process simulations of 183 MOFs. Purity vs. cyclic working capacity is plotted for PSA, TSA– and TSA+ processes, where the color scale represents **a-c** the metal density (g/cm^3) and **d-f** the pore volume (cm^3/g). Symbol size represents the largest cavity diameter (LCD) in Å. The four structures with top performance are named and highlighted, including HKUST-1 (BODPAN), labeled in red. PSA conditions are 298 K, with adsorption at 40 bar and desorption at 1 bar; TSA– conditions are 1 bar, with adsorption at 200 K and desorption at 298 K; TSA+ conditions are 1 bar, with adsorption at 298 K and desorption at 398 K.

S6. Isotherm fitting

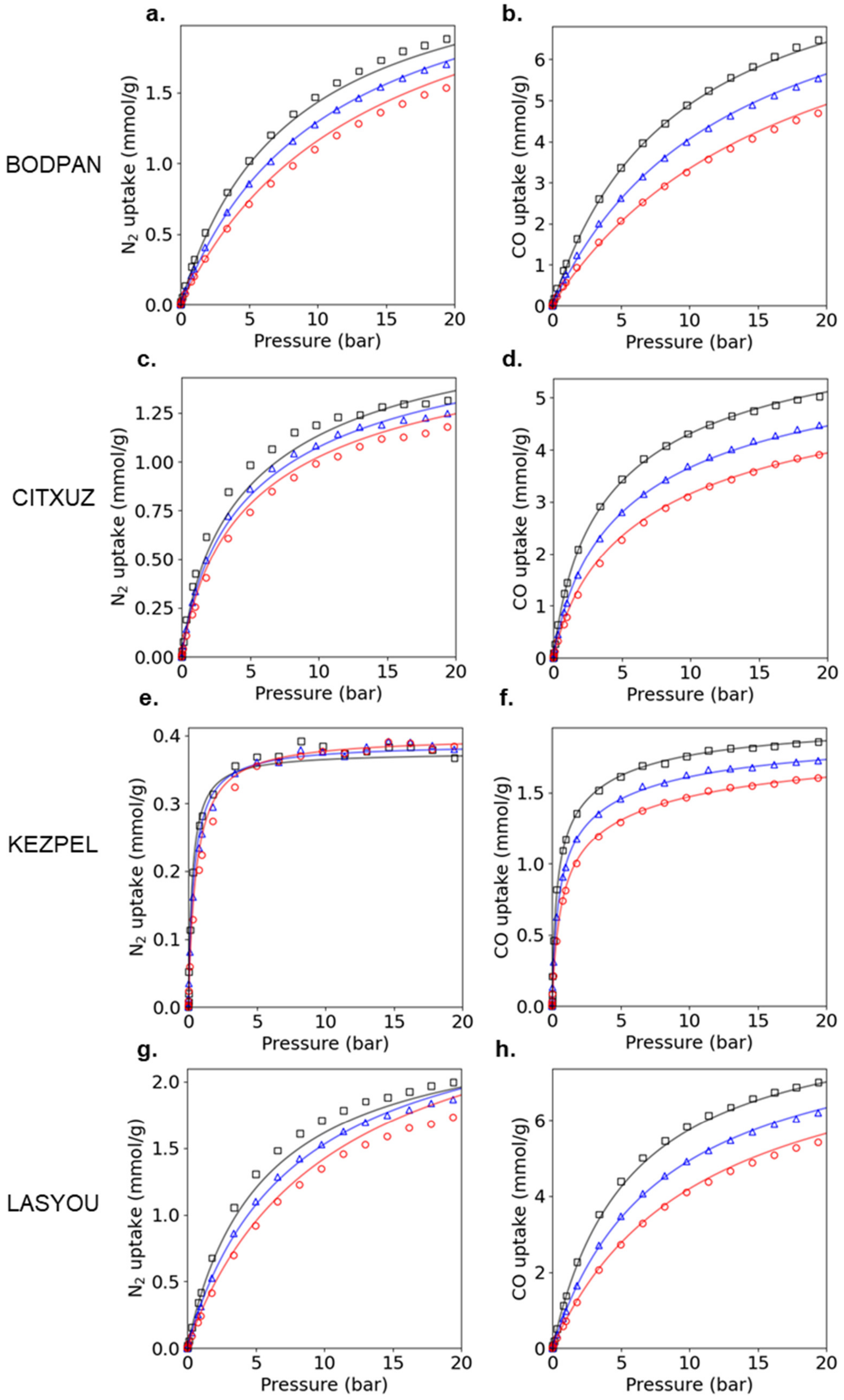

Figure S13 | Comparison of binary mixture DSL model predicted isotherms (lines) and GCMC simulated (symbols) isotherms. N_2 isotherms are in *Column 1* and CO isotherms are in *Column 2*. The isotherms have been computed at 3 different temperatures, 283 K (black and squares), 298 K (blue and triangles), and 313 K (red and circles) for **(a)** and **(b)** – BODPAN; **(c)** and **(d)** – CITXUZ; **(e)** and **(f)** – KEZPEL; **(g)** and **(h)** – LASYOU.

Table S4 | Dual-site Langmuir isotherm fitting parameters for equimolar CO/N_2 mixture adsorption isotherms of BODPAN, CITXUZ, KEZPEL and LASYOU. Column 1 lists the MOF; Column 2 lists the adsorbate, and Columns 3-8 list the Dual-site Langmuir parameters.

MOF	Gas	$N_{m,1}$ (mol/kg)	$N_{m,2}$ (mol/kg)	b_0 (m^3/mol)	d_0 (m^3/mol)	Q_1 (kJ/mol)	Q_2 (kJ/mol)
BODPAN	CO	8.677	2.245	9.568e-05	2.384e-05	16.58466	17.16406
	N_2	7.362	3.560	4.554e-13	6.675e-04	26.31025	11.55724
CITXUZ	CO	12.833	3.767	9.260e-05	0.00727	17.13927	5.87301
	N_2	0.949	15.649	0.0594	7.076e-05	4.65797	10.62776
KEZPEL	CO	0.633	3.962	1.794e-04	2.973e-04	16.55937	19.4859
	N_2	4.020	0.575	2.466e-20	0.00383	32.44458	14.96218
LASYOU	CO	3.185	10.864	0.00315	1.198e-04	1.42737	16.72775
	N_2	0.069	13.979	0.0377	1.760e-04	7.72860	12.15279

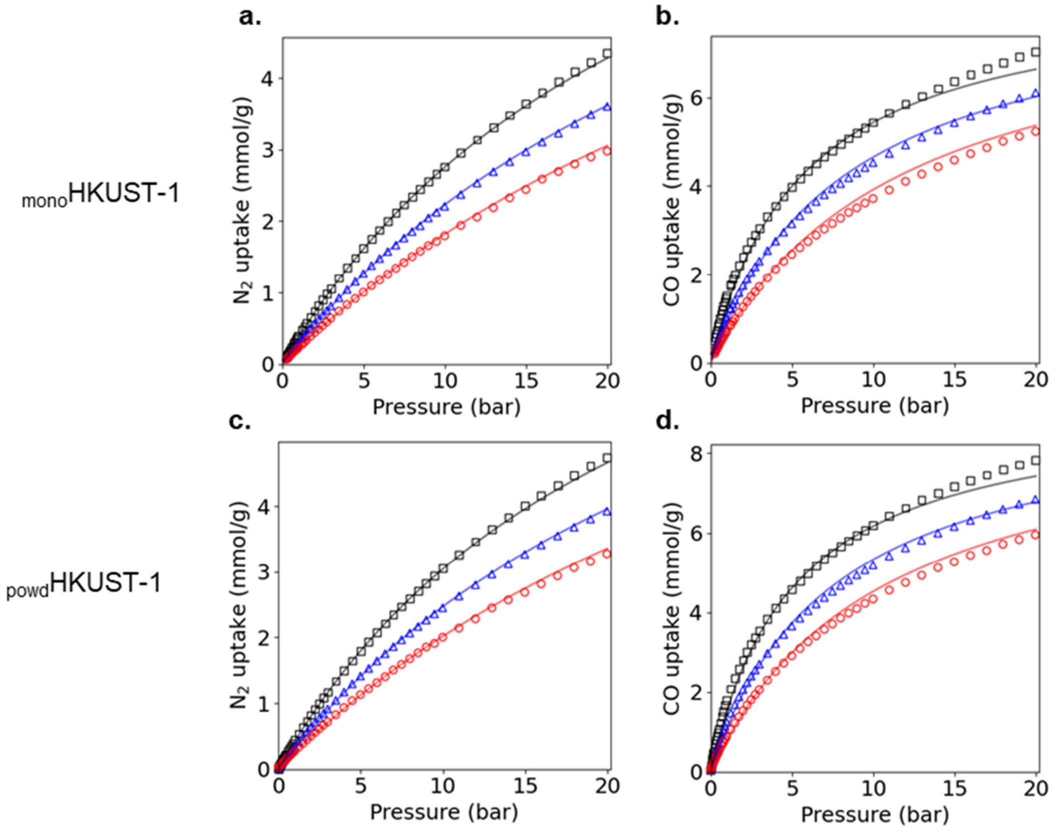

Figure S14 | Comparison of single-component DSL model predicted isotherms (lines) and experimental (symbols) isotherms. N_2 isotherms are in *Column 1* and CO isotherms are in *Column 2*. The isotherms have been computed at 3 different temperatures, 283 K (black and squares), 298 K (blue and triangles), and 313 K (red and circles) for **(a)** and **(b)** – $_{mono}$HKUST-1; **(c)** and **(d) –** $_{powd}$HKUST-1.

Table S5 | Dual-site Langmuir isotherm fitting parameters for single-component adsorption (CO/N_2) isotherms of $_{mono}$HKUST-1 and $_{powd}$HKUST-1. Column 1 lists the MOF; Column 2 lists the adsorbate, and Columns 3-8 list the Dual-site Langmuir parameters.

MOF	Gas	$N_{m,1}$ (mol/kg)	$N_{m,2}$ (mol/kg)	b_0 (m^3/mol)	d_0 (m^3/mol)	Q_1 (kJ/mol)	Q_2 (kJ/mol)
$_{mono}$HKUST-1	CO	0.001	8.521	0.007711	8.869e-05	5.28992	17.87223
	N_2	0.848	9.557	3.329e-07	0.00014	11.36425	13.35611
$_{powd}$HKUST-1	CO	0.225	9.325	0.000126	9.691e-05	0.73258	17.90333
	N_2	9.976	1.866	0.00014	2.792e-06	13.45663	6.94489

S7. References

1. Martin-Calvo, A., F.D. Lahoz-Martin, and S. Calero, *Understanding Carbon Monoxide Capture Using Metal Organic Frameworks.* Journal of Physical Chemistry C, 2012. **116**(11): p. 6655-6663.
2. Potoff, J.J. and J.I. Siepmann, *Vapor–liquid equilibria of mixtures containing alkanes, carbon dioxide, and nitrogen.* AIChE Journal, 2001. **47**(7): p. 1676-1682.
3. Widom, B., *Some topics in the theory of fluids.* The Journal of Chemical Physics, 1963. **39**(11): p. 2808-2812.
4. Leach, A.R., *Molecular modelling: principles and applications*. 2001: Pearson education.
5. Myers, A. and P. Monson, *Adsorption in porous materials at high pressure: theory and experiment.* Langmuir, 2002. **18**(26): p. 10261-10273.
6. Willems, T.F., et al., *Algorithms and tools for high-throughput geometry-based analysis of crystalline porous materials.* Microporous and Mesoporous Materials, 2012. **149**(1): p. 134-141.
7. Brunauer, S., P.H. Emmett, and E. Teller, *Adsorption of Gases in Multimolecular Layers.* Journal of the American Chemical Society, 1938. **60**(2): p. 309-319.
